# Contextual Barriers and Enablers to Safewards Implementation in Victoria, Australia: Application of the Consolidated Framework for Implementation Research

**DOI:** 10.3389/fpsyt.2021.733272

**Published:** 2021-11-04

**Authors:** Justine Fletcher, Lisa Brophy, Jane Pirkis, Bridget Hamilton

**Affiliations:** ^1^Centre for Mental Health, Melbourne School of Population and Global Health, The University of Melbourne, Parkville, VIC, Australia; ^2^School of Allied Health, Human Services and Sport, La Trobe University, Melbourne, VIC, Australia; ^3^Centre for Psychiatric Nursing, School of Health Sciences, The University of Melbourne, Parkville, VIC, Australia

**Keywords:** Safewards, implementation science (MeSH), inpatient mental health services, restrictive practices, seclusion and restraint reduction

## Abstract

**Background:** Safewards is a complex psychosocial intervention designed to reduce conflict and containment on inpatient mental health units. There is mounting international evidence of the effectiveness and acceptability of Safewards. However, a significant challenge exists in promising interventions, such as Safewards, being translated into routine practice. The Consolidated Framework for Implementation Research (CFIR) provides a framework through which to understand implementation in complex health service environments. The aim was to inform more effective implementation of Safewards using the CFIR domains and constructs, capitalizing on developing an understanding of variations across wards.

**Method:** Seven Safewards Leads completed the Training and Implementation Diary for 18 wards that opted in to a trial of Safewards. Fidelity Checklist scores were used to categorize low, medium and high implementers of Safewards at the end of the 12-week implementation period.

**Results:** Qualitative data from the diaries were analyzed thematically and coded according to the five CFIR domains which included 39 constructs. Twenty-six constructs across the five domains were highlighted within the data to have acted as a barrier or enabler. Further analysis revealed that six constructs distinguished between low, medium, and high implementing wards.

**Discussion:** Our findings suggest that for implementation of Safewards to succeed, particular attention needs to be paid to engagement of key staff including managers, making training a priority for all ward staff, adequate planning of the process of implementation and creating an environment on each inpatient unit that prioritize and enables Safewards interventions to be undertaken by staff regularly.

## Introduction

Over the past two decades there has been a growing recognition of the need for improvement in inpatient care delivered to people with serious mental illness (1–6). High acuity, due to low bed numbers and increasing numbers of people being admitted under involuntary treatment orders, creates a challenging environment where consumers are often distressed. Incidents of aggression may be common as well as staff resorting to coercive measures (7, 8). Various forms of aggression (physical, verbal or sexual) and other behaviors of concern (medication refusal, absconding, self-harm) have collectively been termed incidents of conflict (8). Coercive measures and restrictive practices such as seclusion and restraint, are collectively known as containment. Research has shown that consumers commonly report how experiencing or witnessing containment makes them feel unsafe and retraumatised and interferes with their personal recovery and engagement with services (9–13). A smaller body of research has also identified negative impacts for staff who use restrictive practices, whereby they feel guilty using containment methods but also trapped into working this way, due to organizational priorities of managing risk (14–16).

International and national research (17–20) and policy (21–23) over the past two decades underscores the necessity to reduce the use of restrictive practices in inpatient settings. In parallel, there is recognition that translation of practice improvements and implementation of evidence-based practice is challenging (24).

Safewards is one example of a psychosocial intervention that has been developed to reduce conflict and containment and improve mental health care in these settings more generally. Safewards is a theoretical model and set of 10 interventions, outlined in Table 1, which are designed to reduce conflict and containment, thereby improving the safety of consumers and staff (8). The Safewards model suggests that six *originating domains* (the patient community, patient characteristics, regulatory framework, staff team, physical environment, outside hospital) potentially contribute to *flashpoints* (e.g., situations signaling and preceding a conflict event such as physical aggression) which may then lead to *conflict and containment* (26). Under the Safewards model, each of the interventions should be supported by a member of the ward team often known as an “intervention champion.”

**Table 1:** Description of the 10 Safewards interventions (25).

**Safewards intervention**	**Description**
Clear mutual expectations	Involves negotiation process between nurses and consumers, resulting expectations are displayed in a poster
Soft words	Encourages deliberate use of consumer–centered language by nurses, encouraged *via* a set of signs/framed statements, one displayed prominently in staff space and changed frequently
Talk down	Is a structured de-escalation approach, supported by champion role modeling and individually mentoring staff; key elements are displayed in a poster
Positive words	Structures every nursing handover to include positive comments about each consumer
Bad news mitigation	Involves staff sharing at handover any knowledge about consumer experience of bad news or potential events (e.g., denied leave), making priority of listening to consumer concerns when this happens
Know each other	Requires every-day introductory information about each staff member and each consumer to be displayed in a folder, poster or similar for all people in the ward to read
Mutual help meeting	Is a daily or frequent facilitated ward meeting structured to encourage the sharing of thanks, support and requests between consumers
Calm down methods	Provides a set of resources for sensory self-soothing (such as herbal tea, blankets, soft toy, iPods with music, stress balls) freely available for consumers in the ward
Reassurance	Requires the deliberate rounding by nurses to explain and provide support to every consumer who may have been impacted specifically after a conflict event in the ward
Discharge messages	Involves collecting and displaying in the ward encouraging messages from consumers as they leave to ward to other consumers

A cluster randomized controlled trial (cRCT) of Safewards found the model and 10 interventions significantly reduced conflict and containment (27). Later real-world studies of the efficacy of Safewards have demonstrated more mixed results. Some have shown changes to conflict and containment events (25, 28–30), but others have not (31). Findings have highlighted challenges to the implementation of Safewards and identified this as a factor in the range of outcomes (32, 33).

Safewards is not alone here—many interventions are shown to be efficacious in trials but have at best mixed evidence of effectiveness when they are scaled up. Despite research demonstrating the effectiveness of evidence-based interventions, translation into a variety of contexts often fails to flourish and thus improvements in consumer outcomes are lagging behind research evidence (34, 35). Psychosocial interventions, such as Safewards, have been noted to face consistent challenges in uptake in routine service delivery (36). Therefore, growing emphasis has been placed upon the science of implementation (37, 38). Implementation science is the study of techniques used to support the systematic uptake of evidence-based practices into routine practice (39). To date, none of the research into Safewards has provided a detailed evaluation of implementation.

The Consolidated Framework for Implementation Research (CFIR) is one approach to understanding the implementation of complex innovations like Safewards in health care settings (34). CFIR is a meta-theoretical framework based on 19 theories, comprising five domains and 39 constructs known to influence the process of implementation (Table 2) (34). The five domains encompass broad areas and within each domain a series of constructs provides more specific drivers that are known to impact innovation implementation. Not all domains and constructs will be relevant to every innovation (34) the notations next to each construct in Table 2 indicate which were relevant to the present study.

**Table 2:** Domains and associated constructs of the consolidated framework for implementation research.

**Domains/Constructs and subconstructs**	**Short description**
**I. Innovation characteristics**	
A. Intervention source^#^	Perception of key stakeholders about whether the intervention is externally or internally developed
B. Evidence strength and quality^#^	Stakeholders' perceptions of the quality and validity of evidence supporting the belief that the intervention will have desired outcomes
C. Relative advantage^#^	Stakeholders' perception of the advantage of implementing the intervention vs. an alternative solution
D. Adaptability (core components and adaptable periphery) ^#^	The degree to which an intervention can be adapted, tailored, refined, or reinvented to meet local needs
E. Trialability	The ability to test the intervention on a small scale in the organization (8), and to be able to reverse course (undo implementation) if warranted
F. Complexity	Perceived difficulty of implementation, reflected by duration, scope, radicalness, disruptiveness, centrality, and intricacy and number of steps required to implement
G. Design quality and packaging^#^^*^	Perceived excellence in how the intervention is bundled, presented, and assembled
H. Cost	Costs of the intervention and costs associated with implementing that intervention including investment, supply, and opportunity costs
**II. Outer setting**	
A. Needs and resources of those served by the organization	The extent to which patient needs, as well as barriers and facilitators to meet those needs are accurately known and prioritized by the organization
B. Cosmopolitanism^#^	The degree to which an organization is networked with other external organizations
C. Peer pressure	Mimetic or competitive pressure to implement an intervention; typically because most or other key peer or competing organizations have already implemented or in a bid for a competitive edge
D. External policy and incentives^#^	A broad construct that includes external strategies to spread interventions including policy and regulations (governmental or other central entity), external mandates, recommendations and guidelines, pay-for-performance, collaboratives, and public or benchmark reporting
**III. Inner setting**	
A. Structural characteristics^#^	The social architecture, age, maturity, and size of an organization
B. Networks and communications	The nature and quality of webs of social networks and the nature and quality of formal and informal communications within an organization
C. Culture^#^	Norms, values, and basic assumptions of a given organization
D. Implementation climate	The absorptive capacity for change, shared receptivity of involved individuals to an intervention and the extent to which use of that intervention will be rewarded, supported, and expected within their organization
D.1 Tension for change^#^	The degree to which stakeholders perceive the current situation as intolerable or needing change
D.2 Compatibility^#^^**^	The degree of tangible fit between meaning and values attached to the intervention by involved individuals, how those align with individuals' own norms, values, and perceived risks and needs, and how the intervention fits with existing workflows and systems
D.3 Relative priority^#^^*^	Individuals' shared perception of the importance of the implementation within the organization
D.4 Organizational incentives and rewards	Extrinsic incentives such as goal-sharing awards, performance reviews, promotions, and raises in salary and less tangible incentives such as increased stature or respect
D.5 Goals and feedback	The degree to which goals are clearly communicated, acted upon, and fed back to staff and alignment of that feedback with goals
D.6 Learning climate^#^^*^	A climate in which: (a) leaders express their own fallibility and need for team members' assistance and input; (b) team members feel that they are essential, valued, and knowledgeable partners in the change process; (c) individuals feel psychologically safe to try new methods; and (d) there is sufficient time and space for reflective thinking and evaluation
E. Readiness for implementation	Tangible and immediate indicators of organizational commitment to its decision to implement an intervention
E.1 Leadership engagement^#^^**^	Commitment, involvement, and accountability of leaders and managers with the implementation
E.2 Available resources^#^	The level of resources dedicated for implementation and on-going operations including money, training, education, physical space, and time
E.3 Access to knowledge and information^#^^*^	Ease of access to digestible information and knowledge about the intervention and how to incorporate it into work tasks
**IV. Characteristics of individuals**	
A. Knowledge and beliefs about the Intervention^#^^**^	Individuals' attitudes toward and value placed on the intervention as well as familiarity with facts, truths, and principles related to the intervention
B. Self-efficacy^#^	Individual belief in their own capabilities to execute courses of action to achieve implementation goals
C. Individual stage of change	Characterization of the phase an individual is in, as he or she progresses toward skilled, enthusiastic, and sustained use of the intervention
D. Individual identification with organization	A broad construct related to how individuals perceive the organization and their relationship and degree of commitment with that organization
E. Other personal attributes	A broad construct to include other personal traits such as tolerance of ambiguity, intellectual ability, motivation, values, competence, capacity, and learning style
**V. Process**	
A. Planning^#^^*^	The degree to which a scheme or method of behavior and tasks for implementing an intervention are developed in advance and the quality of those schemes or methods
B. Engaging (local training)^#^^*^	Attracting and involving appropriate individuals in the implementation and use of the intervention through a combined strategy of social marketing, education, role modeling, training, and other similar activities
B.1 Opinion leaders^#^^*^	Individuals in an organization who have formal or informal influence on the attitudes and beliefs of their colleagues with respect to implementing the intervention
B.2 Formally appointed internal Implementation Leaders^#^	Individuals from within the organization who have been formally appointed with responsibility for implementing an intervention as coordinator, project manager, team leader, or other similar role
B.3 Champions^#^^**^	“Individuals who dedicate themselves to supporting, marketing, and ‘driving through' an [implementation]” [101] (p. 182), overcoming indifference or resistance that the intervention may provoke in an organization
B.4 External change agents	Individuals who are affiliated with an outside entity who formally influence or facilitate intervention decisions in a desirable direction
B.5 Key stakeholders^#^^*^	Individuals from within the organization that are directly impacted by the innovation, e.g., staff responsible for making referrals to a new program or using a new work process
B.6 Innovation participants^#^^**^	Individuals served by the organization that participate in the innovation, e.g., patients in a prevention program in a hospital
C. Executing^#^^**^	Carrying out or accomplishing the implementation according to plan
D. Reflecting and evaluating^#^^*^	Quantitative and qualitative feedback about the progress and quality of implementation accompanied with regular personal and team debriefing about progress and experience

# *Denotes that the construct was found in our data to represent either an enabler, barrier or mix of both*.

* *Denotes that the construct distinguished **weakly** between low, medium or high implementing wards*.

** *Denotes that the construct distinguished **strongly** between low, medium or high implementing wards NB short descriptions quoted from additional file 3 (34) and CFIR Code Book https://cfirguide.org/tools/tools-and-templates/*.

Damschroder et al. (34) suggest the CFIR can be used in evaluations of implementation at all stages of research design, data collection, and analysis. Consideration of factors that influence implementation generally occurs in one of three ways: (a) specific data are collected relating to the CFIR domains and constructs at the same time as the innovation is being implemented; (b) specific data are collected after the innovation has been implemented either *via* interviews or surveys; or (c) post-evaluation, data that was collected during the implementation of an innovation are analyzed, utilizing the CFIR domains and constructs as a lens to explain the results of implementation. CFIR has been used in these ways to consider barriers and enablers regarding the implementation of innovations (40–42) and to shed light on differences between high and low implementers (41, 43, 44). Using indicators of implementation success is vital when applying CFIR post-evaluation to contextualize any barriers or enablers that are described (34). In turn, this knowledge can be used to further enhance implementation and sustainability of the same and new innovations in routine practice.

The Victorian Safewards Trial (the trial) collected data related to process, impact, and outcome of Safewards implementation. We identified high, medium, and low levels of implementation of Safewards across 18 mental health wards in the Australian state of Victoria, using a fidelity measure designed for Safewards (25, 27). In the current study, we identified levels of implementation across sites and applied the CFIR post-evaluation, to understand the barriers to and enablers of implementation of Safewards in these 18 wards. Our aim was to inform more effective implementation of Safewards using the CFIR domains and constructs, capitalizing on variations across wards. Our specific objectives were:

1. To identify barriers to and enablers of implementing Safewards, based on the CFIR domains and constructs.
2. To determine whether particular CFIR domains and constructs distinguish between high, medium, and low implementers of Safewards.

## Methods

We retrospectively applied the CFIR domains and constructs to process and outcome data that were collected as part of the evaluation of the trial during 2015.

### Study Setting

In 2014, 18 inpatient mental health units representing seven health services opted into the trial funded by the Victorian Government. This equates to one third of the services in Victoria that deliver public mental health services. The trial included adolescent (*n* = 3), adult (*n* = 10), and aged acute wards (*n* = 3), as well as Secure Extended Care Units (*n* = 2) (SECUs) in metropolitan and regional Victoria.

### The Evaluation of Safewards in Victoria

We conducted an independent evaluation of the trial which consisted of three phases, as described in a previous paper by our evaluation team (45). The first was a training phase (November 2014–February 2015) (45) and the second was a 12-week implementation phase (March–May 2015). The third was a sustainability phase (June 2015–April 2016) involving continued fidelity monitoring and outcome measurement, reported elsewhere (25). Each of the health services had one person as the designated Safewards Lead (henceforth referred to as Leads) for the duration of the training and implementation phases.

This paper reports on the the 12-week implementation phase and the application of the CFIR domains and constructs to an analysis of the data collected during that time.

### Ethics Approval

Ethics approval was provided by the University of Melbourne Human Ethics Sub-Committee (ID 1443604), as well as Victorian Human Research Ethics Multi-site (ID 15225L) approval for each of the seven health services that were involved.

### Data Sources and Collection

Implementation data were collected from three sources: (a) a Readiness Checklist; (b) a Fidelity Checklist; and (c) a Training and Implementation Diary. Each of these is described below.

#### Readiness Checklist

The Readiness Checklist collected information pertinent to planning the implementation of each of the 10 Safewards interventions (46). Questions relate to three scales: (a) training (the extent to which training is complete); (b) champions (the appointment of intervention champions); and (c) preparation (the extent to which preparation of materials for each intervention is complete). Leads completed the Readiness Checklist for each of the 18 wards and submitted it to our evaluation team in the week prior to the trial phase. The three scales on the Readiness Checklist were scored out of 10 (one point for each intervention that was prepared).

#### Fidelity Checklist

The Fidelity Checklist is a brief standardized audit tool used by the UK Safewards trial team. It measures the degree to which each intervention has been implemented as intended (27). The tool was completed following a “walk- through” of the ward by evaluation team members, during which observations and discussions with staff were used to compete the checklist. Our evaluation team conducted four walk-throughs of each ward, spending 30–60 mins each time completing the quantitative and qualitative items in the fidelity checklist. These occurred during the trial in March 2015 (Time 1) and May 2015 (Time 3), immediately post-implementation in June 2015 (Time 4), and again during the sustainability phase in March 2016 (Time 6). Times 2 (April 2015 trial phase) and 5 (January 2016 sustainability phase) were conducted by the Leads. The Fidelity Checklist was scored out of 10 to reflect the number of interventions that were being implemented (25).

#### Training and Implementation Diary

Leads were issued with a training and implementation diary consisting of 11 sections (one for the Safewards model and one for each of the Safewards interventions). They were asked to comment in the diaries on the barriers to and enablers of training and implementation in each section. All seven Leads completed the diary for the 18 wards. Where more than one ward in a health service was part of the trial, the Lead consulted with each ward's Safewards intervention Champions to complete the diary. The diary was completed throughout the 4 months of training and implementation and submitted at the end of the 12-week implementation period.

### Data Analysis

Quantitative and qualitative data from the three sources were analyzed at the ward level and mapped to relevant CFIR domains and constructs. All quantitative and qualitative data were coded and rated using the CFIR code book, which presents inclusion and exclusion criteria and examples for each construct (47). An inductive approach (48) was utilized first, for the qualitative data from the training and implementation diaries and the observations recorded in the fidelity checklist, characterizing phenomena that impacted on implementation. Coded data were then theoretically analyzed (49) by mapping barriers and enablers across the CFIR constructs and domains. Data analysis was managed using Nvivo Version 11 (50).

We defined three general levels of readiness that emerged from the Readiness Checklist data. These were: (a) “well prepared” (a score of 7 or above); (b) “somewhat prepared” (a score from 3 to 6); and (c) “under prepared” (a score between 0 and 2).

To assess the implementation of Safewards we used the Time 3 Fidelity Checklist, administered at the end of the 12-week implementation period. Wards were divided into one of three implementation categories based on the Fidelity Checklist score: (a) high implementer (8–10/10); (b) medium implementer (5–7/10); and (c) low implementer (1–4/10).

One member of our team (JF) closely read and inductively coded the qualitative data from the Training and Implementation Diaries and Fidelity Checklists, then theoretically mapped these to the five CFIR domains and their associated 39 constructs, and then further inductively coded the data as either a barrier to or an enabler of implementation for each of the 18 wards. Another member of our team (BH) independently coded a sample of the data and consensus was obtained.

#### Rating CFIR Domains and Constructs

The coded data mapped to each of the CFIR domains and constructs was tabulated per ward and assigned a valence rating. If the content of the coded data demonstrated a positive influence on implementation, this was denoted by ‘+,' and a negative influence was denoted by ‘–.' If data were mixed or equivocal, this was denoted by ‘+/–,' and if it had a neutral impact it was coded as ‘0.' If there were no data for a particular construct, this was regarded as missing (denoted with ‘m'). Next, the strength of the influence was rated as strong (denoted by ‘2') or weak (denoted by ‘1'). The tabulation of data was conducted by one of our team members (JF) and a sample was checked and agreed by a another (BH).

The quantitative data from the Readiness Checklist scales mapped specifically to the following constructs in the CFIR Process domain: (a) *Readiness training*—‘Engaging'; (b) *Readiness champions*—‘Engaging Champions'; and (c) *Readiness preparation*—‘Planning.' The Fidelity Checklist mapped to the Process domain construct of ‘Executing.' Levels of Readiness and Fidelity were transposed to ratings as described above, consistent with published CFIR studies (51, 52) (Table 3).

**Table 3:** Readiness and fidelity checklist scores and the related CFIR rating of valence and strength.

**Data source**	**Range of scores**	**CFIR rating of valence and strength**
**Readiness checklist**		
Prepared	7–10	+2
Somewhat prepared	3–6	+1
Under prepared	0–2	0
**Fidelity checklist**		
High implementation	8–10	+2
Medium implementation	5–7	+1
Low implementation	1–4	0

The CFIR constructs with no data for any ward were omitted., To achieve Objective 1, the for each construct the number of wards was tallied according to each of the following descriptors: (a) an enabler, (b) a barrier, or (c) mixed. This was represented graphically.

To achieve Objective 2, a table was created, in which data from the Fidelity Checklists were used to characterize each ward as being a low, medium, or high implementer. This enabled interrogation of the qualitative data both by construct and by ward implementation level. The data were scrutinized to determine which constructs were weak or strong in terms of discriminating between the levels of implementation. Illustrative quotes are used throughout the results.

## Results

### Objective 1: To Identify Barriers to and Enablers of Implementing Safewards, Based on the CFIR Domains and Constructs

**Figure 1:**
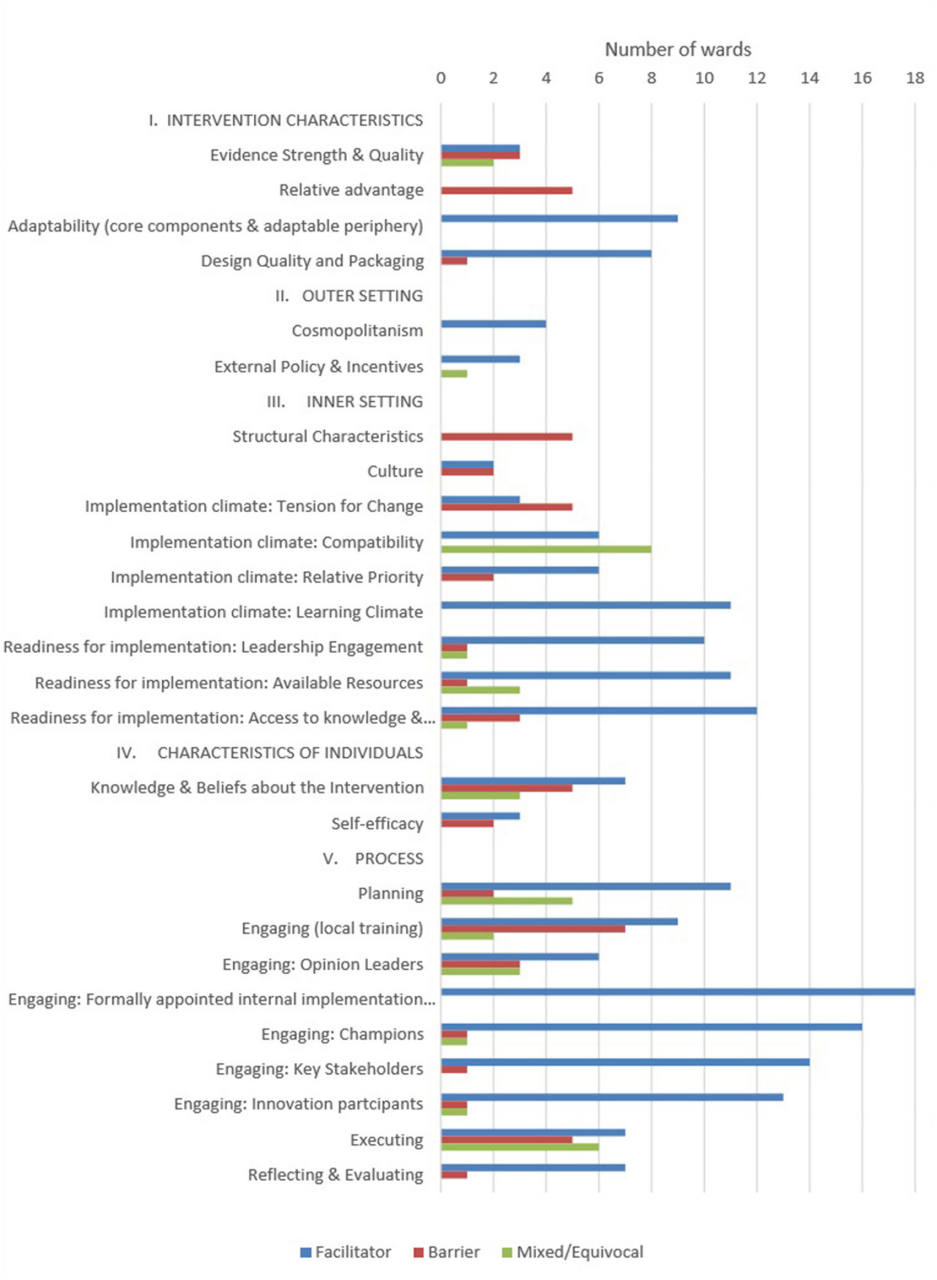
Number of wards highlighting CFIR constructs as barriers or enablers to Safewards implementation.

Twenty-six of the 39 constructs were deemed to be a barrier to or enabler of the implementation of Safewards. Figure 1 illustrates the number of wards in which each of the 26 constructs was an enabler or a barrier to their local implementation. On average, 11 wards contributed data to each of the 26 constructs (range: four wards for three of the constructs to all 18 wards for five constructs). The Process domain constructs with data from all wards were linked to quantitative Readiness and Fidelity Checklist data: Engaging, Engaging Champions, Planning and Executing. Another construct from the Process domain, ‘Engaging: Formally Appointed Internal Implementation Leaders' (B.2), was coded as an enabler for all wards, given the Victorian Government funded a Lead for each ward. Some illustrative quotes are provided as examples of the barriers and enablers highlighted by the leads for some of the constructs.

#### Intervention Characteristics Domain

The Leads were mixed in their reported view of the evidence for Safewards. For some “*easy access to online information and literature reviews”* demonstrated the construct ‘Evidence Strength and Quality' was an enabler. However for others it was a barrier, as illustrated below:

“*aggregate data presented about the benefits of Safewards, individual consumer feedback and experience not presented from the evaluations carried out in the UK.”*

Still others had mixed views about the construct ‘Evidence Strength and Quality.' Some did not perceive value in implementing Safewards compared to another solution, reflecting the construct ‘Relative Advantage.' For example, some Leads reported that staff believed the interventions were reflective of standard practice and therefore didn't value Safewards, as illustrated by the following quote: “*Feelings expressed by staff that intervention is already occurring as part of standard practice.”* Two constructs in the Intervention Characteristics domain that were highlighted by the Leads as enablers were ‘Adaptability' and ‘Design Quality and Packaging.' One Lead stated:

“*We now have a TV on the wall which has most of the profiles uploaded and playing continuously. This has been helpful in ensuring the profiles do not go missing and has also helped alleviate some of the staff anxieties.”*

This finding demonstrates the ‘Adaptability' of the specific intervention ‘Know Each Other.' Leads valued the ‘Design Quality and Packaging' noting that the presentation of training material was excellent and that implementation were well supported:

“*Enabler: The training provided by the Department of Health and the resources including the videos online provided from the UK.”*

#### Outer Setting Domain

The constructs of ‘Cosmopolitanism' and ‘External Policy and Incentives' were each reported as enablers by Leads from a small number of wards. For example Leads valued the opportunity to learn from other health services:

“*Using the examples from other organizations for the displaying of the Talk Down Methods poster to allow for maximum visual effectiveness.”*

Further, “*the funding provided by DHHS and the encouragement to contact the staff from DHHS if required/queries”* were viewed as enablers to implementation.

#### Inner Setting Domain

‘Structural Characteristics' and ‘Implementation Climate: Tension for Change' were more commonly described as barriers than enablers to implementation. Busy wards with high acuity were highlighted by some Leads as barriers to implementation, as described in the following quote:

“*The identified champions for respective interventions felt that they could not find time due to acuity and competing priorities to be able to run more in-services and also to complete some of the work required for their respective interventions.”*

With regard to the construct ‘Implementation Climate: Tension for Change,' the following quote demonstrates the perceptions of some Leads about the staff views:

“*Staff (including medical staff) thinking that they already do debrief consumers involved in incidents and provide support to those who witness incidents (however this is not usually evidenced in the clinical file).”*

‘Implementation Climate: Compatibility' was an enabler in some wards but a mix of barrier and enabler in others. ‘Implementation Climate: Learning Climate' was an enabler. Three sub-constructs of “Readiness for Implementation” were most commonly reported as enablers by Leads, they were ‘Leadership Engagement,' ‘Available Resources,' and ‘Access to Knowledge and Information.'

#### Characteristics of Individuals Domain

Across wards, the construct of ‘Knowledge and Beliefs about the Intervention' was an enabler for some wards, a barrier for others, and for others still it was mixed. ‘Self-Efficacy' was mentioned as both an enabler to implementation and a barrier, as indicated below:

“*Staff report feeling validated that their thoughts on the expectations of the ward can be put together as a group, visual and referred to. This attitude has impacted on the effectiveness of the implementation.”*

In contrast the Lead of another health service reported that

“*A small group of staff displayed minimal understanding of sensory modulation so as a result were unsure of the philosophy being Calm Down Methods and reported feeling not confident in it's application.”*

#### Process Domain

All constructs in the Process domain were highlighted by Leads. Engaging in training was an enabler for some health services, as highlighted by the following quote:

“*The full day training certainly helped the trainers/facilitators deliver the training as suggested at the Train The Trainer sessions. Staff were not pressured for time due to other priorities as they had the full day to complete the training. Staff were keen to attend and some were flexible to attend training on days they would have been rostered off so that this would not impact heavily on ward staffing.”*

However, for other services that took a different approach to training, there were barriers as explained by one Lead: “*The time of year, acuity of wards and sick leave made accessing staff for training difficult.”*

Engaging ‘Key Stakeholders' and ‘Innovation Participants' were enablers. In contrast, engaging ‘Opinion Leaders' within services was a mixed barrier and enabler across wards and within some wards. These two quotes demonstrate the range of experience regarding opinion leaders across health services:

“*Resistive attitude of the medical staff regarding boundaries and disclosure of personal information.”*“*The Relieving Psychiatrist participated in this training and was so taken by the Safewards approach she helped promote it. She read all the handouts and changed her interviewing practices as she just recognized how the Bad News (i.e., No leave, no discharge, increased medication etc) contributed to Flashpoints.”*

‘Planning' was not complete by all wards and was therefore a barrier to those wards.

### Objective 2: To Determine Whether Particular CFIR Domains and Constructs Distinguish Between High and Low Implementers of Safewards

Table 4 provides a matrix comparing CFIR constructs by ward highlighting low, medium, and high implementing wards. Of the 26 constructs to which the data were coded, 15 distinguished between the low, medium, and high implementing wards in this study. Six constructs distinguished strongly across the Inner Setting, Characteristics of Individuals, and Process Domains. Nine constructs were weakly distinguishing from the Intervention Characteristics, Inner Setting, and Process Domains. For the six strongly distinguishing constructs, illustrative quotes from the data along with the description of results are provided below.

**Table 4:** CFIR constructs that distinguish between wards with low and/or medium and/or high Safewards implementation fidelity.

	**Low implementers**					**Medium implementers**						**High implementers**							**Distinguishing construct**
	**1**	**2**	**3**	**4**	**5**	**6**	**7**	**8**	**9**	**10**	**11**	**12**	**13**	**14**	**15**	**16**	**17**	**18**	
**Intervention characteristics**																			
Design quality and packaging	m	+1	m	m	+2	m	m	m	−1	+2	+1	m	m	+1	+2	m	+2	+2	Weak
**Inner setting**																			
Implementation climate: compatibility	0	–*/+*	–*/+*	–*/+*	m	m	–*/+*	m	–*/+*	+2	–*/+*	+2	–*/+*	–*/+*	+1	+2	+2	+2	Strong
Implementation climate: relative priority	m	m	−1	m	m	m	m	+1	+1	m	+1	m	−1	+1	+1	m	m	+1	Weak
Implementation climate: learning climate	m	m	m	m	m	+1	m	+1	+1	+2	m	+1	+1	+2	+2	+1	+2	+1	Weak
Readiness for implementation: leadership engagement	m	m	−1	m	–*/+*	+1	m	+1	+1	+2	+1	+2	+1	m	m	+1	+2	+2	Strong
Readiness for implementation: access to knowledge and information	+1	+1	+1	−1	–*/+*	+1	+1	m	m	+2	−1	+1	−1	+2	+2	+2	+2	+1	Weak
**Characteristics of individuals**																			
Knowledge and beliefs about the intervention	m	−1	–*/+*	–*/+*	+2	−1	−1	+1	−1	+2	m	–*/+*	–*/+*	−1	+2	+1	+2	+2	Strong
**Process**																			
**Planning**	0	0	+1	+2	+2	+2	+1	+2	+2	+2	+2	+1	+2	+1	+2	+1	+2	+2	Weak
**Engaging (local training)**	0	0	+2	0	+2	0	+2	0	0	+2	+2	+2	0	+2	+1	+1	+2	+2	Weak
Engaging: Opinion leaders	–*/+*	−1	–*/+*	m	–*/+*	+1	−1	+1	+1	m	+1	+1	−1	m	m	m	m	+1	Weak
Engaging: Champions	+2	–*/+*	+2	+2	+2	+2	0	+2	+2	+2	+2	+2	+2	+2	+2	+2	+2	+2	Strong
Engaging: Key stakeholders	+1	+2	+1	−1	0	+1	+2	m	m	+2	+2	+1	+1	+2	+2	+1	+2	+2	Weak
Engaging: Innovation participants	m	+1	+1	−1	0	+1	+1	+1	+1	+2	+1	+1	−1,+1	+1	+1	m	+2	+1	Strong
**Executing**	0	0	0	0	0	+1	+1	+1	+1	+1	+1	+2	+2	+2	+2	+2	+2	+2	Strong
**Reflecting and evaluating**	m	+1	+1	m	m	m	m	m	m	+2	+1	m	−1	+2	m	m	+2	+1	Weak

*As described in the CFIR code book (47). **Valence** is depicted as the positive (+) or negative (–) influence the coded data has on the implementation process. Where comments are mixed positive and negative influences on implementation and a clear decision between the two cannot be determined this mix is denoted with –/+. A neutral effect on implementation is denoted with “0” and “m” denotes missing data. **Strength** of the coded component on the implementation of Safewards was rated a strong “2” or weak “1”*.

#### Strongly Distinguishing Constructs

##### Inner Setting Domain

‘Implementation Climate: Compatibility': Data from high implementing wards indicated that Safewards was highly compatible with the current values of staff on the ward and fits well with existing workflow and systems. One Lead from a high implementing ward commented:

“*[Ward] has a dedicated Arts Program which assisted staff to understand how this can also be maximized to work with patients of [ward] to produce discharge message craft pieces for the discharge message tree.”*

A contrasting comment from the Lead in a low implementing ward described how some of the interventions were not aligned to the values and norms of the staff on the ward:

“*Sense of apprehension expressed by some nursing staff about disclosing personal details to consumers. Expectations from the model to include photographs, staff last name etc. for Know Each Other.”*

‘Readiness for Implementation: Leadership Engagement': The medium and high implementing wards revealed strong support for Safewards implementation from ward management, and in some cases hospital management. The following quotation illustrates this:

“*Having members of our senior management/executive team participate in the intervention (Know Each Other) and ongoing support from General Manager / Director of Mental Health”*

This contrasted with statements made by Leads from low implementing wards. Quotations like the following demonstrate a lack of engagement:

“*ANUM [Associate Nurse Unit Manager] team not actively involved with Positive Words, Discharge Messages or Bad News Mitigation. There was a team meeting suggested [by the Lead] with the ANUM team to discuss how this could be utilized better but this was not taken up.”*

##### Characteristics of Individuals Domain

‘Knowledge and Beliefs About the Intervention': Staff of the high implementing wards had a positive attitude and saw value in implementing Safewards, as suggested by the following quotation:

“*Staff are very keenly implementing Safewards and all the interventions, they are showing a great deal of creativity to make Safewards work well.”*

By contrast, staff from the medium and low implementing wards were more mixed in their attitudes. The following comment from the Lead in a low implementing ward illustrates this:

“*Allied health, post grad and graduate nurses have embraced the interventions and Safewards concepts, senior staff much less so, and senior staff have also been reluctant to attend any training or discussions on Safewards.”*

##### Process Domain

‘Engaging: Champions': This construct was first coded according to the Readiness Checklist, and detailed whether a champion was allocated within each ward prior to the start of implementation (16 wards received +2 due to having a champion). However, these codes were then amended where appropriate, based upon feedback from the Leads. Champions of wards in the high implementing group demonstrated commitment and drive to ensure the intervention they were responsible for was successful, as the following comment illustrates:

“*It was decided that the two champions of KEO [Know Each Other] intervention would need to have the attributes of leadership, persistence and a belief in the benefits of this intervention for the long term. This has been very effective, and the champions are well equipped with the resources from the [government]. The champions have contacted the Safewards Lead for the organization at various times to discuss any issues and provide feedback. The KEO champions have worked hard to ensure that the patients of [Ward] have an opportunity to complete the KEO template with great success.”*

By contrast, for some low implementing wards Leads reported delays in engaging champions and noted this was a barrier to implementation. For example, one Lead described their difficulty in “engaging a champion” for the Know Each Other intervention and “*getting an appropriate champion from nursing group for the Calm Down Methods.”*

‘Engaging: Innovation Participants': Leads from the medium and high implementing groups more frequently reported successful attempts to engage consumers on the wards, as the quotation below highlights.

“*Due to a number of patients from [Ward] being at the unit for an extended period of time, the Activity Officer was able to work collaboratively with the patients that are very familiar with the unit… This has assisted greatly in the implementation of this intervention, has given the patients and staff, as reported, a sense of unity and drive to continue with the success of this intervention.”*

‘Executing': This construct was coded based on the fidelity score for each ward. Those wards in the high implementation group were implementing between 8 and 10 Safewards interventions, whereas in the low implementing wards only 3–5 interventions were being implemented.

#### Weakly Distinguishing Constructs

In addition to highlighting the strongly distinguishing constructs above, a brief description of the results for the weakly distinguishing constructs follows. These results may indicate constructs that are important to the implementation of Safewards.

##### Intervention Characteristics Domain

Leads from high implementing wards were more satisfied than the Leads in the low/medium implementing group with the ‘Design Quality and Packaging' of Safewards materials and training materials provided by the government sponsor.

##### Inner Setting Domain

There was a shared perception of the importance of implementing Safewards among the medium/high implementing wards compared to the low implementing wards, indicating ‘Implementation Climate: Relative Priority' was a weak distinguishing construct. As with ‘Implementation Climate: Learning Climate,' data related to this construct was largely missing from the low implementing wards, but between the medium/high implementing wards it was a distinguishing construct. High implementing wards were reportedly environments where staff knowledge was valued, they felt safe to ask questions and share concerns.

Low/medium implementing wards Leads noted a lack of staff being released to attend Safewards training highlighting the construct ‘Readiness for Implementation: Access to Knowledge and Information' distinguished weakly between these wards and high implementing wards.

##### Process Domain

The ‘Planning' construct weakly distinguished between low implementers and the medium/high implementers. Reports on the Readiness Checklist showed some wards had not sourced the materials they required to implement some of the Safewards interventions by the first week of the implementation phase as expected. In addition, there was an active approach taken in some of the medium/high implementing wards to discuss Safewards and its implementation with staff during team meetings, prior to the commencement of training and implementation.

‘Engaging: Opinion Leaders' was also a strength for medium/high implementers. For example, there was positive involvement from consumer consultants and some medical staff noted by Leads. In contrast, low implementing wards experienced resistance from medical staff to be involved.

Leads of medium/high implementing wards commented on the value of ‘Engaging: Key Stakeholders' for training and implementation of specific interventions, for example collaborating with allied health staff for Mutual Help Meetings, Calm Down Methods, and Discharge Messages. In medium/high implementing wards, key staff took responsibility for interventions and this improved implementation.

In some medium/high implementing wards, Leads and managers had taken on the responsibility of ‘Reflecting and Evaluating' on the progress of implementation and had made changes to the implementation based on their observations of things that were not going so well. For example, a unit manager consulted with staff and consumers to produce a first draft of Clear Mutual Expectations, after other attempts had not succeeded.

## Discussion

Our study identified the barriers to and enablers of implementing Safewards, based on the CFIR domains and constructs. To address Objective 1, we coded 26 constructs as implementation enablers, barriers or a mix of both, within and across wards. Nine constructs from the Inner Setting and Process domains were found to be the strongest enablers of implementation (10+ wards). A further four constructs were viewed as enablers by nine or fewer wards, from the domains Intervention Characteristics and Outer Setting.

The mix of views observed between and within wards in our study concurs with the varied reports of Safewards success being related to staff perceptions of the compatibility and relative advantage of implementing Safewards, as opposed to practice as usual or another intervention (33, 53, 54).

Two constructs from the Outer Setting Domain were highlighted by a small number of Leads as being important to implementation. The first ‘Cosmopolitanism,' which describes the link staff from within the ward have to groups outside the organization, was an enabler. As part of the trial, a community of practice was established and the implementation of Safewards in the 18 wards was supported by the government sponsor, which arranged and funded a 3-day train-the-trainer workshop, provided wards with training packs for local training and offered funding to employ a Lead and purchase equipment and print materials. These ‘External Policies and Incentives' were alluded to in some Training and Implementation Diaries as also being an enabler to implementation. However, two Training and Implementation Diaries revealed the implementation timeframe allocated by the government was unrealistic and placed too much pressure on wards that were understaffed and experiencing high staff turnover. This criticism concurs with reports from other research (33, 36).

Further comparison of implementation success to meet Objective 2 revealed that the constructs from the Inner Setting domain were important influencers of the degree of success in implementing Safewards. James et al. (54) concluded implementation of Safewards was low where the intervention was seen to be at odds with the ward structure and flow. This finding indicates the importance of involving frontline staff in the planning and training for Safewards, to create a unified vision of the potential benefits of Safewards, whilst providing a culture of open questioning and learning from leadership staff. This process was demonstrated in the successful implementation of Safewards in one forensic mental health service, using co-creation principles to training and implementation (53).

Other constructs from the Inner Setting Domain underscored that a positive ‘Implementation Climate' was directly related to the ward's readiness for implementation. In low implementing wards in our study there was some obstruction or ambivalence of leadership staff, which resulted in the implementation of Safewards faltering. This aligns with studies showing that lack of strong leadership from ward managers resulted in staff being unclear if Safewards was a priority and gave them license to resist implementation (33, 54). In contrast, the medium and high implementing wards were well supported by strong ‘Leadership Engagement,' demonstrated by unit managers who took responsibility for aspects of implementation, supported champions to undertake their role and created an expectation among staff that Safewards was valued. The existence of a ‘Culture' and ‘Implementation Climate' that supports the implementation and shows ‘Readiness for Implementation' are observed most often in conjunction with successful implementation (38, 43, 54).

The data regarding ‘Characteristics of Individuals' was limited in this study, but when it was available it showed that staff ‘Knowledge and Beliefs' about Safewards and ‘Self-Efficacy' were mixed. The staff in high implementing wards were more likely to display a positive attitude and understanding about Safewards and place value on its implementation, for the benefit of both staff and consumers. The opposite appeared to be true for low and medium implementing wards. This effect has also been demonstrated in other studies (31, 33, 54) suggesting that staff values and knowledge has a direct impact on Safewards implementation.

Importantly, eight of the Process domain constructs underscored differences in implementation success. ‘Engaging: Champions' who are effective and supporting involvement of ward consumers (‘Engaging: Innovation Participants') were strong positive features of medium and high implementing wards. Further ‘Planning' training and implementation, ‘Engaging: Opinion Leaders' within the wards and other ‘Key Stakeholders' such as hospital executives distinguished weakly between low and medium/high implementing wards. Other successful Safewards implementation studies placed great emphasis on the planning of implementation and training (28, 53). This was tailored by using service specific examples and valued by organizations that released staff to attend training and provided fill-in staff (28, 53). Furthermore, results from these studies show ward staff and managers saw the value of Safewards and were knowledgeable and cohesive as a team, during their implementation to a high level of fidelity (28, 53). These features also distinguished between successful and unsuccessful Safewards implementers in the original cRCT conducted in the UK (54). The current study further highlights the importance of involving innovation participants. This was a strongly distinguishing feature between low and medium/high implementers in our study. Two successful implementation studies mentioned the value of involving consumers in their successful implementation (28, 54), whereas lack of consumer involvement was seen as problematic to implementation in another study (31).

### Future Implementation of Safewards and Other Interventions

**Figure 2:**
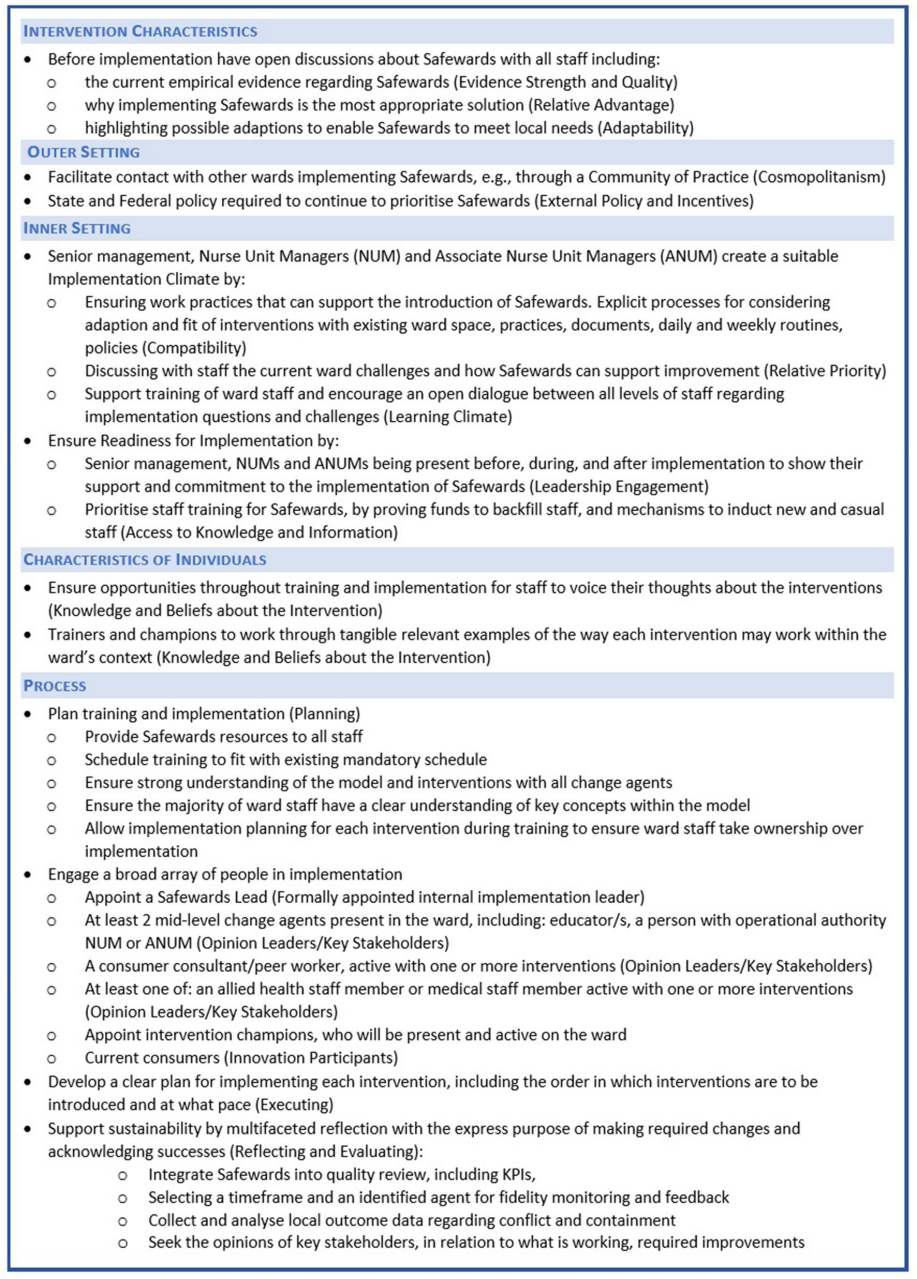
Recommendations for Safewards Implementation based around CFIR domains and constructs.

Our study, together with findings of other studies of Safewards implementation, has demonstrated the complexity of implementation. Hence, we offer recommendations guided by the CFIR domains and constructs that were key barriers and enablers in the Victorian Safewards Trial and specifically those that highlighted successful implementation (see Figure 2). The CFIR framework has been relevant and useful to understanding Safewards implementation.

### Limitations and Future Research

It was not part of our research design to specifically assess implementation based on the CFIR constructs. Therefore, we may have missed some CFIR constructs that were important to the implementation process in these 18 wards. Hence we were reliant on diaries kept by the Leads which were not designed with the CFIR in mind, so we had to treat absence of evidence about a particular construct as evidence of absence. Furthermore ward staff were not asked their views about implementation and their views may have provided important information regarding the constructs that we were unable to report upon.

Future Safewards implementation research would benefit from collecting quantitative and qualitative data from all levels of staff that is directly related to each of the CFIR domains and constructs. In particular regarding the constructs from the Inner Setting and Characteristics of Individuals domains. Given the resistance highlighted in some wards, further consideration of the Innovation Characteristics domain may provide further insight. Specifically, the construct ‘trialability' that related to being able to trial an intervention and reverse it if it doesn't work, may facilitate staff willingness to try something new. Furthermore, understanding the perception of staff regarding the ‘Complexity' of Safewards may offer insight into staff willingness to implement or not.

## Conclusions

Using the CFIR domains and constructs, our study highlighted enablers and barriers at the end of the 12-week implementation phase of Safewards. It found 15 CFIR constructs that distinguished between low, medium and high implementers of Safewards, the majority of which came from the Inner Setting and Process Domains. Our findings offer insight into the important link between these two domains for implementing Safewards. Of particular importance is planning the delivery of training and process of implementation. Further, engagement of a variety of staff who act as champions and opinion leaders; and engagement of innovation participants and key stakeholders who are peripheral or external to the ward, impacts directly on the inner setting. An implementation climate where staff see the compatibility of Safewards with the work they already undertake and the consumers they care for made Safewards a relative priority. When training is enabled and seen to be valued for the whole staff team, this supports a positive learning climate, provides access to resources and the knowledge and information staff require to feel part of the implementation and confident in their role.

## Data Availability Statement

The raw data supporting the conclusions of this article will be made available by the authors, without undue reservation.

## Ethics Statement

This study was conducted in accordance with and after recommendations from Victorian Human Research Ethics Multi-Site Process (ID 15225L). The protocol was approved by Monash Health Human Research Ethics Committee. Additionally ethics approval was obtained from the University of Melbourne Human Ethics Sub-committee (ID 1443604). Written informed consent for participation was not required for this study in accordance with the national legislation and the institutional requirements.

## Author Contributions

JF and BH were involved in the development of the study, data collection, and analysis. JF, BH, and LB were involved in the interpretation of data. All authors were involved in the writing and editing of the manuscript.

## Funding

This paper forms part of the work toward a PhD which was supported through an Australian Government Research Training Program Scholarship. JF was supported by NHMRC PhD Research Scholarship 1133627. This independent evaluation was financially supported by the Office of the Chief Mental Health Nurse, in the Department of Health and Human Services, Government of Victoria.

## Conflict of Interest

The authors declare that the research was conducted in the absence of any commercial or financial relationships that could be construed as a potential conflict of interest.

## Publisher's Note

All claims expressed in this article are solely those of the authors and do not necessarily represent those of their affiliated organizations, or those of the publisher, the editors and the reviewers. Any product that may be evaluated in this article, or claim that may be made by its manufacturer, is not guaranteed or endorsed by the publisher.
